# Assessment of crestal bone loss surrounding dental implants using CBCT in different tissue biotypes

**DOI:** 10.6026/9732063002001179

**Published:** 2024-09-30

**Authors:** Abhaya Chandra Das, Saumyakanta Mohanty, Purobi Choudhury, Priyanka Sarangi, Pallabi Choudhury, Rashmi Rekha Mallick, Saurav Panda, Sindhu Soumya Dash

**Affiliations:** 1 Department of Periodontics and Oral Implantology, Institute of Dental Sciences, Siksha 'O' Anusandhan (Deemed to be University), Khandagiri Square, Bhubaneswar - 751030 Odisha, India; 2 Department of Conservative Dentistry & Endodontics, SCB Dental College & Hospital, Cuttack, Odisha - 753007, India; 3 Department of Dentistry, Silchar Medical College and Hospital, Attached faculty of Government Dental College, Silchar, Department of Periodontics, Srimanta Sankardeva University of Health Science's, Assam, India; 4 Lecturer, Government Dental College, Silchar, Srimanta Sankardeva University of Health Science's, Assam, India; 5 Intern, Kalinga Institute of Dental Sciences, Bhubaneswar, Odisha, India

**Keywords:** Cone beam computed tomography (CBCT), crestal bone loss, dental implant

## Abstract

Marginal bone loss (MBL) is a crucial marker of implant health. Hence this study was done to assess the amount of height of crestal bone lost surrounding dental implants positioned in different tissue types. Twenty six patients with single edentulous areas were divided into two groups at random with 13 samples in each group .Groups A and B comprise implants placed in thick tissue biotypes and thin tissue biotypes, respectively. Cone beam computed tomography (CBCT) was done in both groups at baseline and after implant placement to evaluate the loss of crestal bone surrounding the distal and mesial sides of the implants. Before occlusal loading, at the moment of cementation, a follow-up CBCT was taken. During the time of cementation, both groups experienced a discernible loss of crestal bone on the mesial and distal sides of the implants; however, group B shows a larger loss of crestal bone than group A. Compared to Group A (thick tissue biotype), Group B (thin tissue biotype) had a greater mean crestal bone loss. During the peri-implant healing period, crestal bone alterations are less common in thick biotypes than thin biotypes.

## Background:

Teeth loss is commonly treated with dental implants. Marginal bone loss (MBL) is a crucial marker of implant health. Radiographic pictures are the most important resources for evaluating the MBL surrounding implants. The maximum loading limit for the first year of loading is 1.5 mm, and for each additional year after that, it is 0.2 mm. The development of a black triangle is one of MBL's clinical consequences. Consequently, aesthetics will be compromised if the MBL is more on the mesial side of implant [1]. The success of implants is influenced by several factors, including the kind of prosthesis, crestal bone loss, dental health habits, occlusal loading, surrounding soft tissues and the frequency of reminder visits [2- 3].It has been suggested that in order for a durable epithelial connective tissue attachment to occur, at least 3 mm of peri-implant mucosa should exist. Furthermore, it has been ted that thicker tissues construct the biologic breadth surrounding implants with less bone resorption than thinner tissues [4]. Another name for crestal soft tissue is "vertical soft tissue". The majority of techniques used to evaluate vertical soft tissue breadth till date [5]. A clinical technique for determining the thickness of the crestal soft tissue before placing dental implants was described by Linkevicius *et al.* [6]. Cone-beam computed tomography (CBCT), an-invasive, accurate way to visualise anatomical features has been developed. Because of its excellent resolution and relatively low exposure dose, CBCT has shown to be very helpful in the field of dentistry [7]. Today, a common method for assessing implant success is cone beam computed tomography (CBCT) analysis. CBCT offers crucial information for determining the ultimate implant size and site. When applied to dental implants, CBCT can show the implant's location as well as the surrounding anatomical structure, bone angulation, and the implant's form, contour, and thickness at different heights [8]. Therefore, it is of interest to identify the amount of crestal bone height lost surrounding dental implants positioned with different tissue types.

## Materials and Methods:

The Periodontics and Oral Implantology department conducted this study after taking inclusion and exclusion criteria into account. Participants' informed consent forms were obtained, and the ethical committee's permission was received. This prospective cross sectional study was done form May 2022 to August 2023. Demographic profile of each patient was recorded. Twenty six patients with solitary edentulous sites were alienated into two groups with 13 samples in each group at random: Group A (implants implanted in thick tissue biotype) and Group B (implants placed in thin tissue biotype). A single operator performed all surgical procedures and radiographic measurements. Using an acrylic stent and endodontic reamer, 30, soft tissue thickness was measured 3 mm apical to the crest. A full thickness flap was reflected after a crestal incision was made in the middle of the edentulous ridge using an aseptic technique. The osteotomy was prepared in accordance with the manufacturer's instructions and all implants were inserted in a traditional two-stage process. To determine the mesiodistal space dimensions, the suggested implant site's bone density, and its proximity to neighbouring important structures, a preoperative CBCT scan was performed. Distal and mesial crestal bone height was taken into consideration at baseline and after cementation, both at the distal and mesial side of the implant. Sirona Orthophos SL (Dentsply Sirona, Charlotte, NC, USA) was used for CBCT. The Instrumentarium OP300 was used to capture scans at 89.6 kV, 6 mA, 14 s and 0.3 mm3 voxel sizes. Using 6TM Software (Anatomage, San Jose, CA, version 6.0.4) measurements and analysis were done on vertical cross-section views perpendicular to the alveolar ridge at the middle of each edentulous site. To ensure that the implant guide pin was positioned correctly; an intra-operative radiograph was taken after placement of implant. Baseline CBCT was done in both groups following implant placement, and follow-up CBCT was performed during cementation, just before occlusal loading, to quantify the amount of crestal bone lost in both groups around the mesial and distal sides of the implants. SPSS statistical software version 23.0 (Chicago, IL, USA) and SAS version 9.4 (SAS Institute Inc., Cary, NC) was used with AVA test at p < 0.05 for statistical analysis of the data.

## Results:

Both groups had a considerable loss of crestal bone at the mesial and distal aspects of the implants at the time of cementation, although group B had a greater loss of crestal bone than group A. Although crestal bone loss was statistically negligible, it was somewhat more on the mesial side than the distal. There is little difference between the groups when it comes to crestal bone loss on the mesial or distal side (Table 1, Table 2).

## Discussion:

Dental implants are a cutting-edge treatment option for restoring lost function and aesthetics following tooth extraction. Traditionally, mobility, marginal bone loss and sulcus depth, have been utilised as clinical indicators of implant effectiveness [2]. Alteration in the crestal bone height surrounding dental implants positioned in various tissue biotypes were assessed by Mushtaq *et al.* They came to the conclusion that thick biotypes result in less alterations to the crestal bone than thin biotypes, which induce more crestal bone loss [2]. According to Abdinian *et al.* there were appreciable differences in the marginal bone loss between the maxilla and mandible and mesial and distal implant sides [1]. In their one-year study, Kaminaka *et al.* examined the alterations in soft tissue around implants in thick and thin biotype groups. They found that the thin biotype group had higher CBL than the thick biotype group [9]. Algethmi *et al.* evaluated the reduction in crestal bone height surrounding dental implants placed in diverse tissue biotypes. They came to the conclusion that thin tissue biotypes had a higher mean crestal bone loss than thick tissue biotypes [10]. Our observations are in line with these results. Cui*et al.* investigated potential correlations among crestal soft tissue thickness and measures of hard tissue on CBCT images. They concluded that there was discernible relationship between the values of hard tissue and the thickness of crestal soft tissue. Edenticulous anterior sites and maxillary posterior sites showed thicker crestal soft tissue at the alveolar crest in comparison to mandibular posterior locations [5]. Mushtaq *et al.* reported that tissue thickness decreased in both the thick and thin biotype patients from baseline to cementation; however, the thin biotype group experienced a greater decrease in tissue thickness and concluded that initial tissue biotype plays a considerable role in early crestal bone loss surrounding implants [11]. The mean difference in pain and mobility between the groups that received implants immediately after and those that received them later was found to be n-significant by Randhir *et al.* [8]. Moussa *et al.* concluded that, using CBCT in soft tissue assessment at the time of implant/bone evaluation can aid in saving the clinician and patient time and prevents the painful invasive bone sounding process [12]. By employing CBCT analysis to assess the buccal, lingual, distal and mesial crestal bone around the implant, Trivedi *et al.* came to the conclusion that bone loss surrounding the implant crest module is a result of multiple factors, and that maintaining the crestal bone will aid in distributing the functional load [13]. Similarly Deshpande *et al.* concluded from their study that, crestal bone levels around dental implants should be preserved for long-standing accomplishment of dental implants [14]. Crestal marginal bone loss in the most coronal region of one-piece implants is considerably smaller than the marginal bone loss seen in tapered screws, according to research by Aragoneses *et al.* [15]. When evaluating the survival rate of short dental implants in patients with compromised health, Jagadeesh *et al.* came to the conclusion that these individuals had a higher risk of dental implant failure than did healthy subjects [16]. We discovered that Group B (thin tissue biotype) had a higher average crestal bone loss than Group A (thick tissue biotype). The findings must be confirmed by other research.

## Conclusion:

Group B thin tissue biotype had a higher average loss of crestal bone compared to thick tissue biotype. Thick biotype results in fewer crestal bone alterations in contrast to thin biotype, which induces greater crestal bone loss during the peri-implant healing phase.

## Figures and Tables

**Table 1:** Mesial crestal bone loss among groups

**Groups**	**Mean± SD (crestal bone loss)**	P
Group A	0.12± 0.23	0.011
Group B	0.20± 0.43	0.01
Group A vs B	-0.08± 0.20	0.243

**Table 2:** Distal crestal bone loss among groups

**Groups**	**Mean± SD (crestal bone loss)**	P
Group A	0.10± 0.18	0.013
Group B	0.17± 0.48	0.006
Group A vs B	-0.07± 0.30	0.265
